# Supplementary material for: Modeling glioblastoma heterogeneity as a dynamic network of cell states
Source: Mol Syst Biol. 2021 Sep 16;17(9):e10105. doi: 10.15252/msb.202010105 (PMC8444284; doi:10.15252/msb.202010105)
Supplement: Supplementary file 5 — Source Data for Figure 3 [file MSB-17-e10105-s001.zip › Figure3A_sourcedata/GSEA_3065/nosigdb_state2.GseaPreranked.1622718419215/NB_KI_CLUSTER2.html]

Details for gene set NB\_KI\_CLUSTER2[GSEA]

|  || Dataset | state2 |
| Phenotype | NoPhenotypeAvailable |
| Upregulated in class | na\_neg |
| GeneSet | NB\_KI\_CLUSTER2 |
| Enrichment Score (ES) | -0.5461811 |
| Normalized Enrichment Score (NES) | -2.3694994 |
| Nominal p-value | 0.0 |
| FDR q-value | 0.0 |
| FWER p-Value | 0.0 |
Table: GSEA Results Summary

  

Fig 1: Enrichment plot: NB\_KI\_CLUSTER2      
 Profile of the Running ES Score & Positions of GeneSet Members on the Rank Ordered List

  

| PROBE | GENE SYMBOL | GENE\_TITLE | RANK IN GENE LIST | RANK METRIC SCORE | RUNNING ES | CORE ENRICHMENT || 1 | CPNE2 |  |  | 629 | 0.159 | -0.0600 | No |
| 2 | MARCKS |  |  | 630 | 0.159 | -0.0516 | No |
| 3 | VEZF1 |  |  | 882 | 0.130 | -0.0720 | No |
| 4 | UBE2E3 |  |  | 966 | 0.122 | -0.0745 | No |
| 5 | TRIM37 |  |  | 1173 | 0.106 | -0.0913 | No |
| 6 | CXXC5 |  |  | 1185 | 0.105 | -0.0869 | No |
| 7 | HDGFL3 |  |  | 1194 | 0.105 | -0.0822 | No |
| 8 | PPP2CB |  |  | 1355 | 0.095 | -0.0946 | No |
| 9 | TMSB10 |  |  | 1358 | 0.094 | -0.0898 | No |
| 10 | TTL |  |  | 1391 | 0.093 | -0.0883 | No |
| 11 | TCEA2 |  |  | 1501 | 0.086 | -0.0956 | No |
| 12 | PHIP |  |  | 1505 | 0.086 | -0.0914 | No |
| 13 | DEAF1 |  |  | 1680 | 0.078 | -0.1062 | No |
| 14 | PCBD1 |  |  | 1732 | 0.076 | -0.1077 | No |
| 15 | STX2 |  |  | 1741 | 0.075 | -0.1046 | No |
| 16 | PRXL2A |  |  | 1807 | 0.072 | -0.1078 | No |
| 17 | MPP6 |  |  | 1886 | 0.069 | -0.1126 | No |
| 18 | ZEB1 |  |  | 1995 | 0.065 | -0.1209 | No |
| 19 | BRI3 |  |  | 2058 | 0.062 | -0.1244 | No |
| 20 | CNRIP1 |  |  | 2088 | 0.061 | -0.1243 | No |
| 21 | PLEKHO1 |  |  | 2095 | 0.061 | -0.1216 | No |
| 22 | TBCD |  |  | 2125 | 0.060 | -0.1216 | No |
| 23 | RPL7L1 |  |  | 2200 | 0.057 | -0.1266 | No |
| 24 | EIF2AK2 |  |  | 2212 | 0.057 | -0.1248 | No |
| 25 | BRD3 |  |  | 2316 | 0.053 | -0.1332 | No |
| 26 | NR2F6 |  |  | 2376 | 0.051 | -0.1369 | No |
| 27 | CRK |  |  | 2409 | 0.051 | -0.1377 | No |
| 28 | RHBDD2 |  |  | 2451 | 0.049 | -0.1395 | No |
| 29 | SIAH1 |  |  | 2552 | 0.046 | -0.1479 | No |
| 30 | CCDC82 |  |  | 2564 | 0.046 | -0.1467 | No |
| 31 | FSD1 |  |  | 2893 | 0.037 | -0.1805 | No |
| 32 | HIPK2 |  |  | 3029 | 0.033 | -0.1934 | No |
| 33 | DAAM1 |  |  | 3107 | 0.031 | -0.2001 | No |
| 34 | USP11 |  |  | 3142 | 0.031 | -0.2022 | No |
| 35 | SMARCD3 |  |  | 3236 | 0.029 | -0.2107 | No |
| 36 | ZC2HC1A |  |  | 3248 | 0.029 | -0.2104 | No |
| 37 | YPEL1 |  |  | 3286 | 0.028 | -0.2130 | No |
| 38 | GNG2 |  |  | 3367 | 0.026 | -0.2203 | No |
| 39 | CEP290 |  |  | 3418 | 0.025 | -0.2244 | No |
| 40 | SPIN1 |  |  | 3500 | 0.024 | -0.2319 | No |
| 41 | SCRN1 |  |  | 3513 | 0.024 | -0.2320 | No |
| 42 | ENAH |  |  | 3666 | 0.021 | -0.2474 | No |
| 43 | SESTD1 |  |  | 3726 | 0.020 | -0.2527 | No |
| 44 | PCBP4 |  |  | 3779 | 0.019 | -0.2574 | No |
| 45 | RAB3B |  |  | 3834 | 0.018 | -0.2623 | No |
| 46 | FBXO21 |  |  | 3903 | 0.017 | -0.2688 | No |
| 47 | ATXN7L3B |  |  | 4009 | 0.015 | -0.2794 | No |
| 48 | KBTBD6 |  |  | 4014 | 0.015 | -0.2790 | No |
| 49 | CCDC88A |  |  | 4035 | 0.015 | -0.2804 | No |
| 50 | ZNRF1 |  |  | 4070 | 0.014 | -0.2833 | No |
| 51 | TMOD2 |  |  | 4152 | 0.013 | -0.2915 | No |
| 52 | LINC00667 |  |  | 4348 | 0.010 | -0.3122 | No |
| 53 | PPP2R2B |  |  | 4358 | 0.010 | -0.3126 | No |
| 54 | C5orf24 |  |  | 4375 | 0.009 | -0.3139 | No |
| 55 | GSTA4 |  |  | 4418 | 0.009 | -0.3180 | No |
| 56 | MIR4458HG |  |  | 4557 | 0.006 | -0.3327 | No |
| 57 | BTBD3 |  |  | 4597 | 0.006 | -0.3366 | No |
| 58 | ATP6V1B2 |  |  | 4676 | 0.004 | -0.3449 | No |
| 59 | RBM26 |  |  | 4724 | 0.004 | -0.3498 | No |
| 60 | LSP1P5 |  |  | 4726 | 0.004 | -0.3497 | No |
| 61 | KIAA0930 |  |  | 4941 | 0.001 | -0.3730 | No |
| 62 | DIXDC1 |  |  | 4982 | 0.000 | -0.3773 | No |
| 63 | C16orf45 |  |  | 5003 | -0.000 | -0.3795 | No |
| 64 | PJA2 |  |  | 5030 | -0.000 | -0.3823 | No |
| 65 | SFSWAP |  |  | 5048 | -0.001 | -0.3841 | No |
| 66 | ZNF33A |  |  | 5115 | -0.002 | -0.3912 | No |
| 67 | CCZ1 |  |  | 5179 | -0.003 | -0.3979 | No |
| 68 | EFNB2 |  |  | 5207 | -0.003 | -0.4007 | No |
| 69 | APBB1 |  |  | 5288 | -0.005 | -0.4091 | No |
| 70 | RAPGEF6 |  |  | 5326 | -0.005 | -0.4129 | No |
| 71 | CDC42EP4 |  |  | 5335 | -0.005 | -0.4135 | No |
| 72 | SECISBP2 |  |  | 5398 | -0.007 | -0.4198 | No |
| 73 | ZC3H6 |  |  | 5425 | -0.007 | -0.4223 | No |
| 74 | LRRC37B |  |  | 5448 | -0.008 | -0.4243 | No |
| 75 | GNAI1 |  |  | 5503 | -0.009 | -0.4297 | No |
| 76 | PCGF2 |  |  | 5528 | -0.009 | -0.4318 | No |
| 77 | DYNC1I2 |  |  | 5531 | -0.009 | -0.4316 | No |
| 78 | SNAP47 |  |  | 5607 | -0.010 | -0.4392 | No |
| 79 | RALGDS |  |  | 5656 | -0.011 | -0.4438 | No |
| 80 | GSTM3 |  |  | 5666 | -0.011 | -0.4442 | No |
| 81 | ORAI2 |  |  | 5669 | -0.011 | -0.4439 | No |
| 82 | NOL4L |  |  | 5675 | -0.011 | -0.4438 | No |
| 83 | SENP6 |  |  | 5828 | -0.014 | -0.4596 | No |
| 84 | MYCBP2 |  |  | 5830 | -0.014 | -0.4590 | No |
| 85 | TUBGCP4 |  |  | 5854 | -0.014 | -0.4608 | No |
| 86 | TP53BP1 |  |  | 5915 | -0.015 | -0.4665 | No |
| 87 | MAP4K5 |  |  | 5973 | -0.016 | -0.4719 | No |
| 88 | THRA |  |  | 5975 | -0.016 | -0.4711 | No |
| 89 | EPB41L1 |  |  | 5977 | -0.016 | -0.4704 | No |
| 90 | CD200 |  |  | 6001 | -0.017 | -0.4720 | No |
| 91 | ICA1L |  |  | 6013 | -0.017 | -0.4723 | No |
| 92 | CACNB3 |  |  | 6042 | -0.017 | -0.4744 | No |
| 93 | AHI1 |  |  | 6071 | -0.018 | -0.4765 | No |
| 94 | ABR |  |  | 6092 | -0.018 | -0.4777 | No |
| 95 | CLASP2 |  |  | 6110 | -0.018 | -0.4786 | No |
| 96 | SUPT20H |  |  | 6157 | -0.019 | -0.4826 | No |
| 97 | SMARCA1 |  |  | 6258 | -0.021 | -0.4924 | No |
| 98 | VAT1 |  |  | 6300 | -0.022 | -0.4957 | No |
| 99 | DTX3 |  |  | 6311 | -0.022 | -0.4956 | No |
| 100 | PARP6 |  |  | 6317 | -0.022 | -0.4950 | No |
| 101 | TNRC6C |  |  | 6329 | -0.022 | -0.4950 | No |
| 102 | HIST3H2A |  |  | 6335 | -0.022 | -0.4944 | No |
| 103 | EPC1 |  |  | 6397 | -0.024 | -0.4997 | No |
| 104 | RUNDC1 |  |  | 6513 | -0.025 | -0.5109 | No |
| 105 | CCSER2 |  |  | 6522 | -0.026 | -0.5104 | No |
| 106 | TSPYL4 |  |  | 6568 | -0.027 | -0.5139 | No |
| 107 | LPCAT4 |  |  | 6576 | -0.027 | -0.5132 | No |
| 108 | FOXN3 |  |  | 6582 | -0.027 | -0.5123 | No |
| 109 | PLEKHA8 |  |  | 6600 | -0.027 | -0.5127 | No |
| 110 | RSBN1L |  |  | 6643 | -0.028 | -0.5158 | No |
| 111 | TLN2 |  |  | 6682 | -0.029 | -0.5184 | No |
| 112 | FNBP1L |  |  | 6696 | -0.029 | -0.5183 | No |
| 113 | KIAA0232 |  |  | 6710 | -0.030 | -0.5181 | No |
| 114 | MAPRE3 |  |  | 6739 | -0.030 | -0.5195 | No |
| 115 | ZNF429 |  |  | 6757 | -0.031 | -0.5198 | No |
| 116 | FKBP1B |  |  | 6791 | -0.031 | -0.5217 | No |
| 117 | NUDT3 |  |  | 6794 | -0.031 | -0.5202 | No |
| 118 | PNMA1 |  |  | 6837 | -0.032 | -0.5231 | No |
| 119 | TLE4 |  |  | 6872 | -0.033 | -0.5250 | No |
| 120 | IFI27L2 |  |  | 6887 | -0.034 | -0.5247 | No |
| 121 | PRAF2 |  |  | 6920 | -0.035 | -0.5264 | No |
| 122 | ZMYM4 |  |  | 6929 | -0.035 | -0.5254 | No |
| 123 | LARP6 |  |  | 6936 | -0.035 | -0.5242 | No |
| 124 | EPN2 |  |  | 7019 | -0.037 | -0.5311 | No |
| 125 | ARMCX1 |  |  | 7034 | -0.037 | -0.5307 | No |
| 126 | ZDHHC21 |  |  | 7047 | -0.038 | -0.5300 | No |
| 127 | KIF1B |  |  | 7160 | -0.041 | -0.5400 | No |
| 128 | TCF4 |  |  | 7166 | -0.041 | -0.5383 | No |
| 129 | PPP1R9A |  |  | 7239 | -0.043 | -0.5439 | Yes |
| 130 | TMX4 |  |  | 7244 | -0.043 | -0.5420 | Yes |
| 131 | PREPL |  |  | 7249 | -0.043 | -0.5402 | Yes |
| 132 | SRGAP1 |  |  | 7250 | -0.043 | -0.5379 | Yes |
| 133 | DNMT3A |  |  | 7260 | -0.044 | -0.5365 | Yes |
| 134 | ZFHX3 |  |  | 7288 | -0.044 | -0.5371 | Yes |
| 135 | PCNX2 |  |  | 7290 | -0.044 | -0.5348 | Yes |
| 136 | RNMT |  |  | 7302 | -0.045 | -0.5337 | Yes |
| 137 | PRKACB |  |  | 7318 | -0.045 | -0.5329 | Yes |
| 138 | TMCO3 |  |  | 7330 | -0.045 | -0.5317 | Yes |
| 139 | PTPRN2 |  |  | 7358 | -0.046 | -0.5322 | Yes |
| 140 | ATRX |  |  | 7408 | -0.047 | -0.5350 | Yes |
| 141 | LAPTM4B |  |  | 7427 | -0.048 | -0.5344 | Yes |
| 142 | MTURN |  |  | 7440 | -0.048 | -0.5331 | Yes |
| 143 | PSD3 |  |  | 7470 | -0.050 | -0.5336 | Yes |
| 144 | TAOK1 |  |  | 7489 | -0.050 | -0.5329 | Yes |
| 145 | CTTNBP2NL |  |  | 7501 | -0.050 | -0.5314 | Yes |
| 146 | PRRT2 |  |  | 7518 | -0.051 | -0.5305 | Yes |
| 147 | MXI1 |  |  | 7589 | -0.053 | -0.5353 | Yes |
| 148 | GALNT11 |  |  | 7629 | -0.054 | -0.5366 | Yes |
| 149 | GSE1 |  |  | 7639 | -0.054 | -0.5347 | Yes |
| 150 | MAP1LC3A |  |  | 7650 | -0.055 | -0.5329 | Yes |
| 151 | MCC |  |  | 7673 | -0.055 | -0.5324 | Yes |
| 152 | LNPK |  |  | 7730 | -0.057 | -0.5354 | Yes |
| 153 | ZNF397 |  |  | 7735 | -0.057 | -0.5328 | Yes |
| 154 | TET1 |  |  | 7765 | -0.059 | -0.5329 | Yes |
| 155 | SLC39A6 |  |  | 7769 | -0.059 | -0.5301 | Yes |
| 156 | RAB40B |  |  | 7781 | -0.059 | -0.5281 | Yes |
| 157 | PTBP2 |  |  | 7861 | -0.062 | -0.5334 | Yes |
| 158 | PFN2 |  |  | 7867 | -0.062 | -0.5307 | Yes |
| 159 | TSPYL1 |  |  | 7895 | -0.063 | -0.5303 | Yes |
| 160 | DYNC1LI2 |  |  | 7904 | -0.063 | -0.5278 | Yes |
| 161 | CASD1 |  |  | 7906 | -0.063 | -0.5246 | Yes |
| 162 | TUT4 |  |  | 7922 | -0.064 | -0.5228 | Yes |
| 163 | CEP170 |  |  | 7930 | -0.064 | -0.5202 | Yes |
| 164 | HEXD |  |  | 7940 | -0.064 | -0.5177 | Yes |
| 165 | ZNF37A |  |  | 7993 | -0.067 | -0.5198 | Yes |
| 166 | ATP6V1H |  |  | 8025 | -0.068 | -0.5196 | Yes |
| 167 | ARGLU1 |  |  | 8034 | -0.068 | -0.5168 | Yes |
| 168 | PCDH9 |  |  | 8035 | -0.068 | -0.5131 | Yes |
| 169 | NEDD4L |  |  | 8042 | -0.069 | -0.5101 | Yes |
| 170 | LPIN1 |  |  | 8050 | -0.069 | -0.5072 | Yes |
| 171 | CDK5RAP3 |  |  | 8063 | -0.070 | -0.5048 | Yes |
| 172 | UBN2 |  |  | 8070 | -0.070 | -0.5018 | Yes |
| 173 | TRIM16 |  |  | 8146 | -0.074 | -0.5060 | Yes |
| 174 | TULP4 |  |  | 8168 | -0.076 | -0.5043 | Yes |
| 175 | PTPRA |  |  | 8219 | -0.078 | -0.5056 | Yes |
| 176 | TSC2 |  |  | 8259 | -0.080 | -0.5055 | Yes |
| 177 | NPEPPS |  |  | 8268 | -0.081 | -0.5021 | Yes |
| 178 | EXOC4 |  |  | 8300 | -0.083 | -0.5010 | Yes |
| 179 | BPTF |  |  | 8311 | -0.083 | -0.4977 | Yes |
| 180 | ACAP3 |  |  | 8320 | -0.084 | -0.4941 | Yes |
| 181 | DBN1 |  |  | 8326 | -0.084 | -0.4902 | Yes |
| 182 | SPAG9 |  |  | 8330 | -0.084 | -0.4860 | Yes |
| 183 | IFT20 |  |  | 8338 | -0.085 | -0.4823 | Yes |
| 184 | AUTS2 |  |  | 8356 | -0.086 | -0.4796 | Yes |
| 185 | SHC1 |  |  | 8385 | -0.088 | -0.4779 | Yes |
| 186 | TSHZ2 |  |  | 8391 | -0.088 | -0.4738 | Yes |
| 187 | NHLRC3 |  |  | 8397 | -0.089 | -0.4696 | Yes |
| 188 | HPCAL1 |  |  | 8409 | -0.090 | -0.4660 | Yes |
| 189 | SMAD1 |  |  | 8439 | -0.091 | -0.4643 | Yes |
| 190 | MAFG |  |  | 8449 | -0.092 | -0.4604 | Yes |
| 191 | SLC25A36 |  |  | 8496 | -0.095 | -0.4603 | Yes |
| 192 | DPYSL2 |  |  | 8507 | -0.096 | -0.4563 | Yes |
| 193 | MCF2L |  |  | 8514 | -0.097 | -0.4518 | Yes |
| 194 | MEST |  |  | 8534 | -0.099 | -0.4486 | Yes |
| 195 | CASC15 |  |  | 8541 | -0.099 | -0.4439 | Yes |
| 196 | APC |  |  | 8542 | -0.100 | -0.4386 | Yes |
| 197 | ZNF292 |  |  | 8563 | -0.101 | -0.4355 | Yes |
| 198 | PLD3 |  |  | 8576 | -0.102 | -0.4314 | Yes |
| 199 | FAM171B |  |  | 8591 | -0.102 | -0.4274 | Yes |
| 200 | CPEB4 |  |  | 8604 | -0.104 | -0.4232 | Yes |
| 201 | FOXO3 |  |  | 8616 | -0.104 | -0.4189 | Yes |
| 202 | PDE4DIP |  |  | 8665 | -0.108 | -0.4184 | Yes |
| 203 | MSI2 |  |  | 8667 | -0.108 | -0.4127 | Yes |
| 204 | ZNF91 |  |  | 8675 | -0.109 | -0.4077 | Yes |
| 205 | TTLL7 |  |  | 8688 | -0.110 | -0.4031 | Yes |
| 206 | WDR6 |  |  | 8689 | -0.110 | -0.3972 | Yes |
| 207 | FAM215B |  |  | 8711 | -0.112 | -0.3936 | Yes |
| 208 | PRRC2B |  |  | 8734 | -0.115 | -0.3899 | Yes |
| 209 | VEZT |  |  | 8739 | -0.115 | -0.3842 | Yes |
| 210 | PWAR6 |  |  | 8749 | -0.117 | -0.3789 | Yes |
| 211 | CSNK1E |  |  | 8756 | -0.117 | -0.3734 | Yes |
| 212 | PGM2L1 |  |  | 8768 | -0.118 | -0.3683 | Yes |
| 213 | TRIM13 |  |  | 8792 | -0.122 | -0.3643 | Yes |
| 214 | BICD1 |  |  | 8882 | -0.133 | -0.3669 | Yes |
| 215 | MUM1 |  |  | 8888 | -0.134 | -0.3604 | Yes |
| 216 | MAP1A |  |  | 8919 | -0.137 | -0.3563 | Yes |
| 217 | ANKRD12 |  |  | 8935 | -0.139 | -0.3505 | Yes |
| 218 | SLC39A10 |  |  | 8939 | -0.140 | -0.3435 | Yes |
| 219 | PBX1 |  |  | 8940 | -0.140 | -0.3360 | Yes |
| 220 | DSEL |  |  | 8943 | -0.140 | -0.3288 | Yes |
| 221 | ARHGAP21 |  |  | 8949 | -0.141 | -0.3218 | Yes |
| 222 | BAZ2B |  |  | 8963 | -0.143 | -0.3156 | Yes |
| 223 | MTMR4 |  |  | 8973 | -0.145 | -0.3089 | Yes |
| 224 | SORBS2 |  |  | 8978 | -0.145 | -0.3016 | Yes |
| 225 | KDM5B |  |  | 8992 | -0.147 | -0.2952 | Yes |
| 226 | SRGAP3 |  |  | 9014 | -0.151 | -0.2894 | Yes |
| 227 | MAPK10 |  |  | 9041 | -0.156 | -0.2839 | Yes |
| 228 | CHST12 |  |  | 9050 | -0.157 | -0.2764 | Yes |
| 229 | EFHC1 |  |  | 9059 | -0.159 | -0.2688 | Yes |
| 230 | CHD9 |  |  | 9066 | -0.160 | -0.2609 | Yes |
| 231 | EIF4A2 |  |  | 9067 | -0.160 | -0.2524 | Yes |
| 232 | RTN4 |  |  | 9099 | -0.166 | -0.2469 | Yes |
| 233 | DNM1 |  |  | 9109 | -0.168 | -0.2390 | Yes |
| 234 | PNISR |  |  | 9119 | -0.170 | -0.2309 | Yes |
| 235 | ALCAM |  |  | 9132 | -0.173 | -0.2230 | Yes |
| 236 | IP6K2 |  |  | 9136 | -0.174 | -0.2141 | Yes |
| 237 | CLCN3 |  |  | 9179 | -0.185 | -0.2089 | Yes |
| 238 | NPDC1 |  |  | 9188 | -0.186 | -0.1998 | Yes |
| 239 | DYNC1H1 |  |  | 9231 | -0.205 | -0.1935 | Yes |
| 240 | CLSTN1 |  |  | 9246 | -0.210 | -0.1839 | Yes |
| 241 | MARCH6 |  |  | 9247 | -0.211 | -0.1727 | Yes |
| 242 | TANC2 |  |  | 9256 | -0.215 | -0.1621 | Yes |
| 243 | KIDINS220 |  |  | 9260 | -0.217 | -0.1509 | Yes |
| 244 | RETREG1 |  |  | 9262 | -0.218 | -0.1394 | Yes |
| 245 | SESN3 |  |  | 9266 | -0.219 | -0.1281 | Yes |
| 246 | PRH1 |  |  | 9289 | -0.228 | -0.1183 | Yes |
| 247 | GOLM1 |  |  | 9298 | -0.230 | -0.1070 | Yes |
| 248 | ZKSCAN1 |  |  | 9306 | -0.233 | -0.0954 | Yes |
| 249 | AKAP9 |  |  | 9318 | -0.244 | -0.0836 | Yes |
| 250 | KANSL1 |  |  | 9335 | -0.259 | -0.0715 | Yes |
| 251 | NRP2 |  |  | 9357 | -0.281 | -0.0589 | Yes |
| 252 | ANK3 |  |  | 9361 | -0.290 | -0.0438 | Yes |
| 253 | KCNQ1OT1 |  |  | 9414 | -0.434 | -0.0264 | Yes |
| 254 | CLU |  |  | 9430 | -0.547 | 0.0011 | Yes |
Table: GSEA details [plain text format]

  

Fig 2: NB\_KI\_CLUSTER2: Random ES distribution      
 Gene set null distribution of ES for **NB\_KI\_CLUSTER2**
